# An easy gene assembling strategy for light-promoted transfection by combining host-guest interaction of cucurbit[7]uril and gold nanoparticles

**DOI:** 10.1038/s41598-017-06449-9

**Published:** 2017-07-20

**Authors:** Jianwei Du, Peng Zhang, Xiao Zhao, Youxiang Wang

**Affiliations:** MOE Key Laboratory of Macromolecular Synthesis and Functionalization, Department of Polymer Science and Engineering, Zhejiang University, Hangzhou, 310027 P. R. China

## Abstract

Cucurbit[7]uril (CB[7]), a representative member of the host family cucurbit[n]uril, can host-guest interact with many guest molecules such as adamantane, viologen and naphthalene derivatives. This host-guest interaction provides an easy strategy in gene vector assembling. Furthermore, CB[7] can self-assemble on gold nanospheres (AuNSs). Herein, the combination of CB[7] and AuNSs provides both advantages of host-guest interaction and photo-thermal effect of AuNSs. In this study, polyethyleneimine (PEI) and polyethylene glycol (PEG) were separately interacted with CB[7] via host-guest interaction. Then by assembling on AuNSs, PEI and PEG were combined together to condense DNA into polyplexes as well as enhance circulation stability of the polyplexes. These gene vectors were found to have high cellular uptake efficiency and low cytotoxicity. Furthermore, the well distributed AuNSs in the polyplexes could transform light into heat under light exposure because of the photo-thermal effect. This was found to effectively promote the entry of gene into cytoplasm and highly enhanced gene transfection efficiency.

## Introduction

Gene delivery is a promising method for tumor therapy. Host-guest interaction has some advantages in gene and drug delivery. For instance, the process of host-guest interaction is always simply vortexing or sonicating the mixed solution of host molecules and the guest molecules, which is easy-handling and mild compared with chemical reactions. Furthermore, the kind of the ligands as well as their amount can be easily adjusted by changing the kind of the functional ligands and their mole ratios^1–3^. Cucurbit[n]uril is a big family of host molecules. Its hydrophobic cavity can encapsulate many guest molecules such as adamantane, viologen and naphthalene derivatives to form inclusion complexes^4–7^. Compared with other commonly used host molecules such as cyclodextrin (CD), cucurbit[n]uril exhibits stronger interaction and better specificity. For instance, the binding affinities of CB[7] to its guests are between 10^7^ M^−1^ and 10^17^ M^−1^, which can form very stable host-guest inclusion complexes^8–11^. Thus, by simply linking PEG and other functional tags onto cationic polymers via host-guest interaction, multifunctional gene vectors can be easily designed. Unfortunately, most of the CB[n] members are found to have poor water solubility and hard to covalently link to cationic polymers, which restrict their application in gene delivery^12^. However, CB[7] with seven repeated units of glycoluril shows good water solubility and it is interesting to find that CB[7] can self-assemble on gold nanospheres (AuNSs)^13, 14^. This property offers the opportunity to combine host-guest interaction with AuNSs. As is reported, AuNSs has photo-thermal property which can transform light into heat^15, 16^. In this way, gene may be easily released upon the irradiation of light and the heated AuNSs can agitate cell membrane and may promote the direct entry of gene into cytoplasm or the endosome escape. Therefore, light-controlled gene vector can be designed with high gene transfection efficiency based on host-guest interaction.

**Figure 1:**
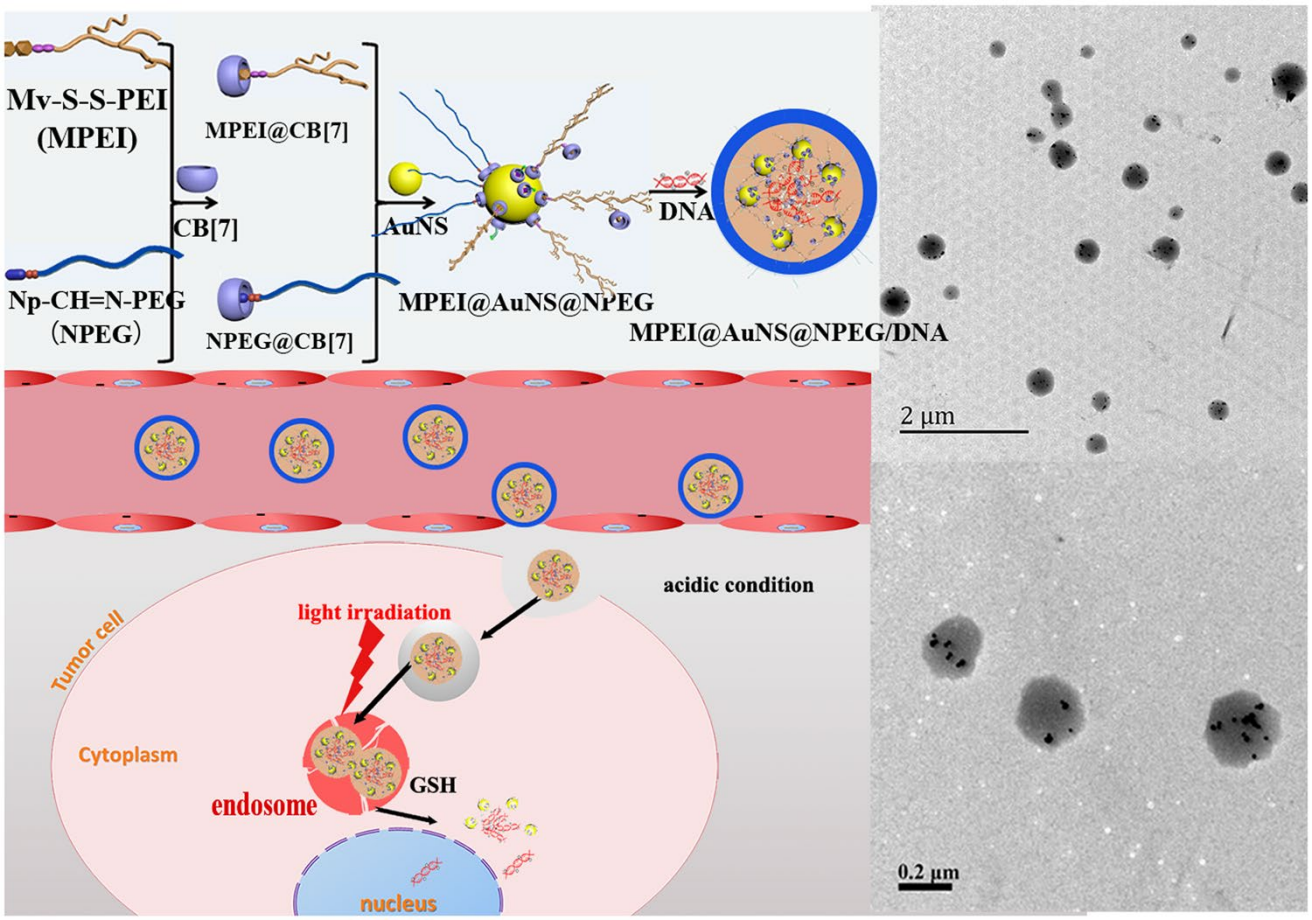
Schematic showing the assembling and delivery process (left) and TEM images (right) of MPEI@AuNS@NPEG/DNA polyplexes.

Herein, we synthesized methyl viologen modified polyethylenimine (Mv-S-S-PEI, MPEI) and naphthalene modified polyethyleneglycol (Np-CH=N-PEG, NPEG) via pH- and GSH-responsive bonds, respectively (Fig. 1). Via host-guest interaction with CB[7], MPEI and NPEG were expected to ‘graft onto’ one side of CB[7]. This would leave the other side of CB[7] to assemble on gold nanosphere (AuNSs). Then the MPEI could electrostatically interact with DNA to form nanoparticles. The resulted gene vectors were expected to be stabilized by both NPEG shell and AuNSs as crosslinking points. While entering into tumor tissue or cells, the pH- and GSH- responsive bonds were expected to break for better DNA release and high cellular uptake efficiency^17–20^. Furthermore, we expected the photo-thermal effect of AuNSs would accelerate the release of DNA and promote endosome escape of gene for higher gene transfection efficiency upon light irradiation. Therefore, gene vector with both advantages of host-guest interaction and AuNSs would be achieved in one shot.

## Results and Discussion

### Synthesis process of MPEI and NPEG

**Figure 2:**
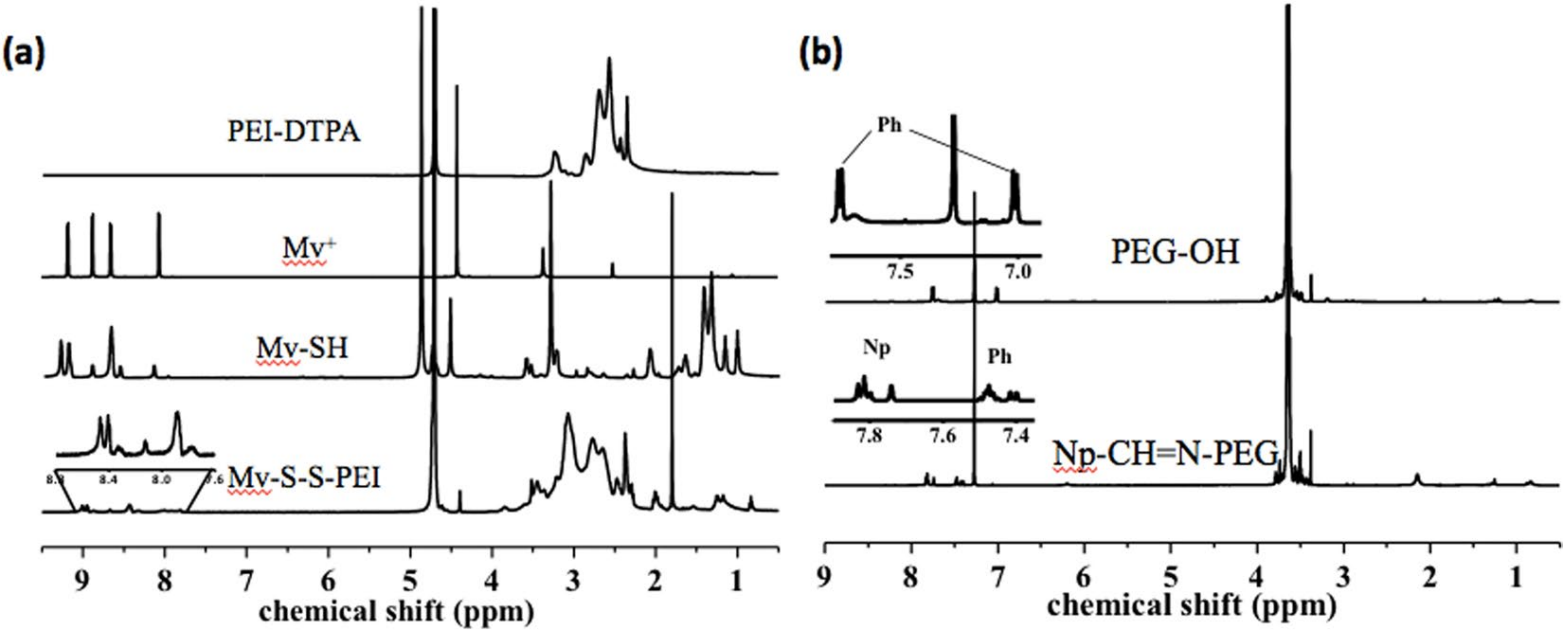
(**a**) ^1^H NMR spectra of Mv-S-S-PEI and corresponding products in corresponding deuterium reagent; (**b**) ^1^H NMR spectra of Np-CH=N-PEG and its corresponding product in CDCl_3_.

As was shown in Fig. 2, Mv-SH was successfully synthesized step by step as reported and confirmed by ^1^H NMR^5, 21^. In order to bond Mv onto PEI with disulfide bond, 3,3′-dithiodipropionic acid (DTPA) was firstly linked to PEI via condensation reaction. Then, -SH were exposed at the presence of tris(2-carboxyethyl) phosphine (TCEP). Because Mv-SH had smaller molecular weight than PEI-SH themselves, they would had higher reactivity and firstly reacted with PEI-SH. By oxidizing the -SH step by step through the addition of O_2_ and H_2_O_2_ (1%), Mv-S-S-PEI (MPEI) was synthesized. The unreacted Mv-SH and other byproducts such as Mv-S-S-Mv were getting rid of via dialysis. Based on the integration ratio of the peaks corresponding to Mv groups (8.42–9.00, 8 H) and PEI (2.05–3.10, -CH_2_CH_2_-NH-), about 9 Mv groups were determined to be bonded to a PEI chain.

At the same time, PEG was modified with the guest molecule naphthalene (Np). 4-hydroxybenzaldehyde was used to connect 1-naphthylacetic acid and PEG-NH_2_ via Schiff base bond^22^. The successful synthesis of Np-CH=N-PEG (NPEG) was also confirmed by the integration ratio between peaks corresponding to Np and Ph groups (7.35–7.88, aromatic, 11 H) and PEG (3.30–3.90, -CH_2_CH_2_-O-). Each NPEG chain was linked with one Np molecule.

### Chemo-physical characterization of different polyplexes

**Figure 3:**
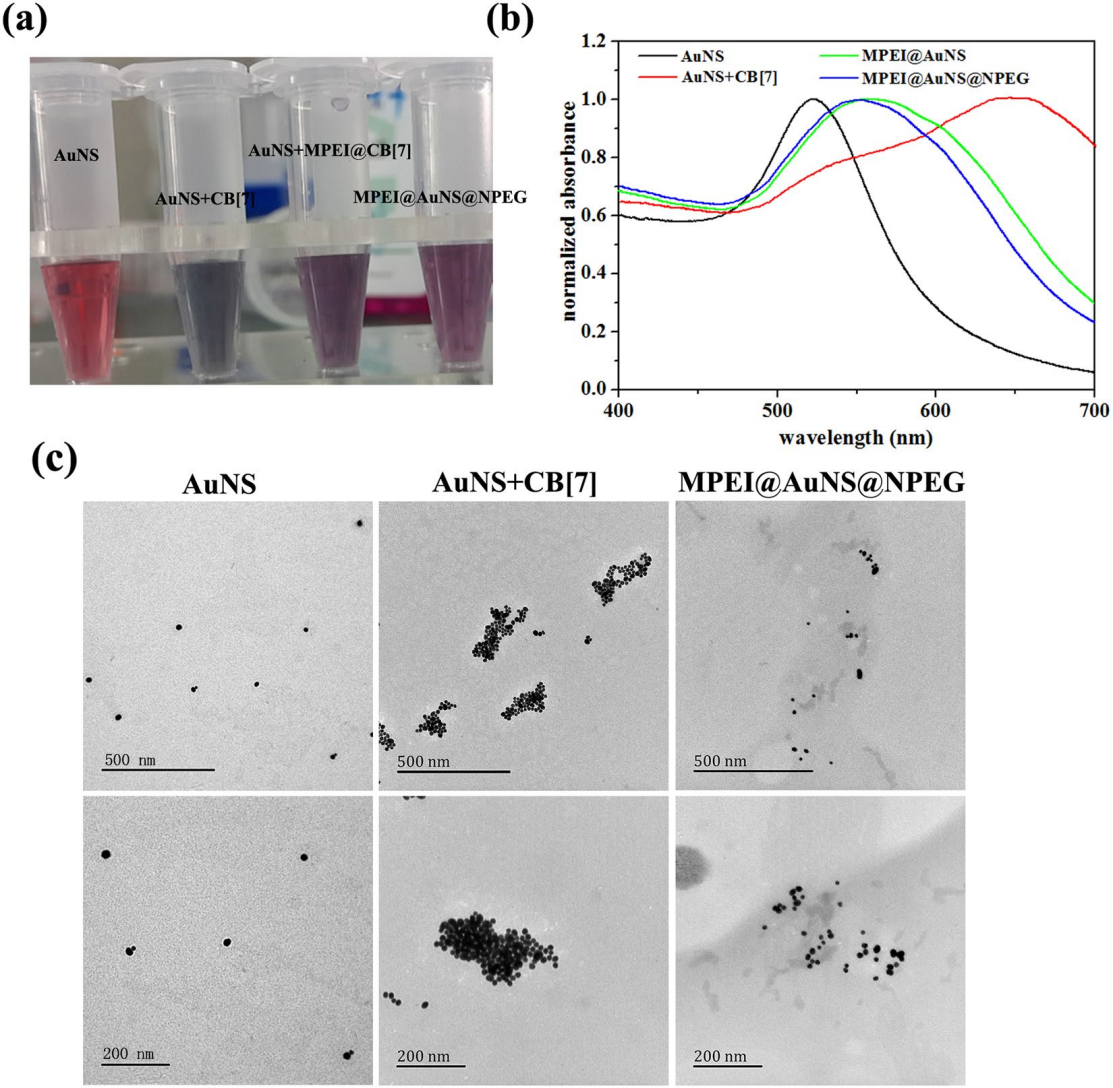
(**a**) Digital photos of AuNS, AuNS + CB[7], AuNS + MPEI@CB[7] and MPEI@AuNS@NPEG solution with the same AuNSs and CB[7] concentration after 2 h incubation; (**b**) UV-Vis spectra of AuNS, AuNS + CB[7], AuNS + MPEI@CB[7] and MPEI@AuNS@NPEG solution with the same AuNSs and CB[7] concentration after 2 h incubation; (**c**) TEM images of different nanoparticles.

As was reported, both sides of CB[7] could interact with AuNSs, which would cause the aggregation of AuNSs^4, 21^. In our system, CB[7] would firstly host-guest interact with MPEI or NPEG, which would leave the other side of CB[7] assembling onto AuNSs. This might finally link MPEI or NPEG onto AuNSs without causing the aggregation of AuNSs. To prove this, AuNSs were interacted with CB[7], MPEI@CB[7] and NPEG@CB[7] and observed by digital photos, UV-Vis spectra as well as TEM images. As was shown in Fig. 3, AuNSs solution was red and had a UV-Vis absorption peak at 525 nm. However, after the addition of CB[7] only, the AuNSs solution turned black with the UV-Vis absorption peak shifting prodigiously to 650 nm. This suggested the aggregation of AuNSs and thus proved the interaction between AuNSs and both sides of CB[7]. However, while MPEI@CB[7] and NPEG@CB[7] were added, the AuNSs solution only became deep red with the absorption shifting a little to long wavelength. This confirmed our presumption that the interaction of MPEI or NPEG with CB[7] left only one side of CB[7] to assemble on AuNSs. Furthermore the little red shifting of UV-Vis absorption would be favourable for photo-thermal conversion^23^. These results were also in accordance with the TEM images. As shown in Fig. 3(c), the aggregation of AuNSs was obviously shown with the addition of CB[7] only, while the AuNSs distributed well with the addition of MPEI@CB[7] and NPEG@CB[7].

After the assembly with AuNSs, the MPEI@AuNS@NPEG solution was vortexed with DNA solution. Via electrostatic interaction with DNA, the DNA polyplexes were formed at different N/P ratios and were designated as MPEI@AuNS@NPEG/DNA. Other polyplexes were named similar with it. N/P ratio referred to the mole ratio between protonatable nitrogen atoms of PEI and the phosphate groups of the DNA.

**Figure 4:**
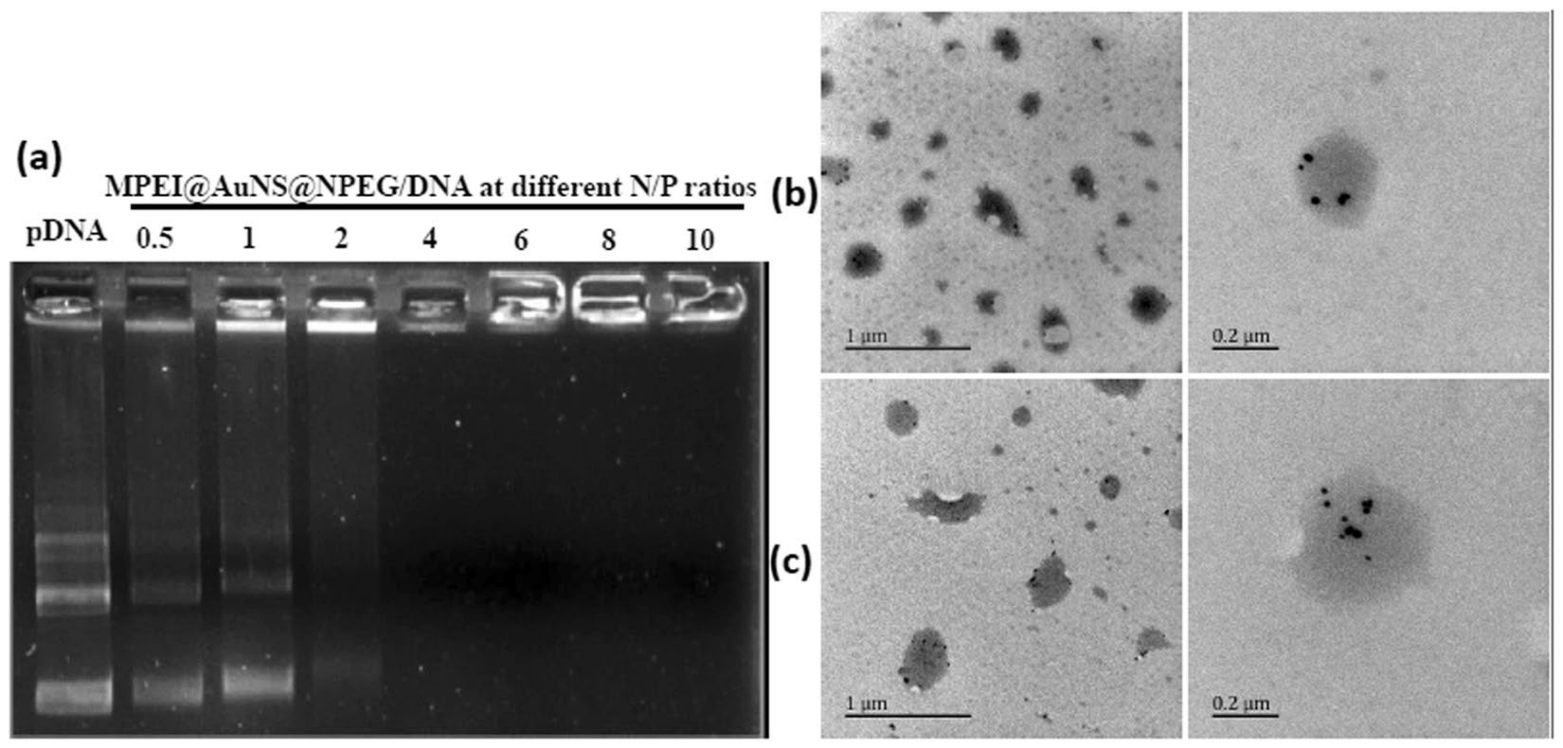
(**a**) Agarose gel retardation assay of MPEI@AuNS@NPEG/DNA polyplexes; (**b**) (**c**) TEM images of MPEI@AuNS@NPEG/DNA polyplexes at N/P ratios of 30 and 50, respectively.

**Figure 5:**
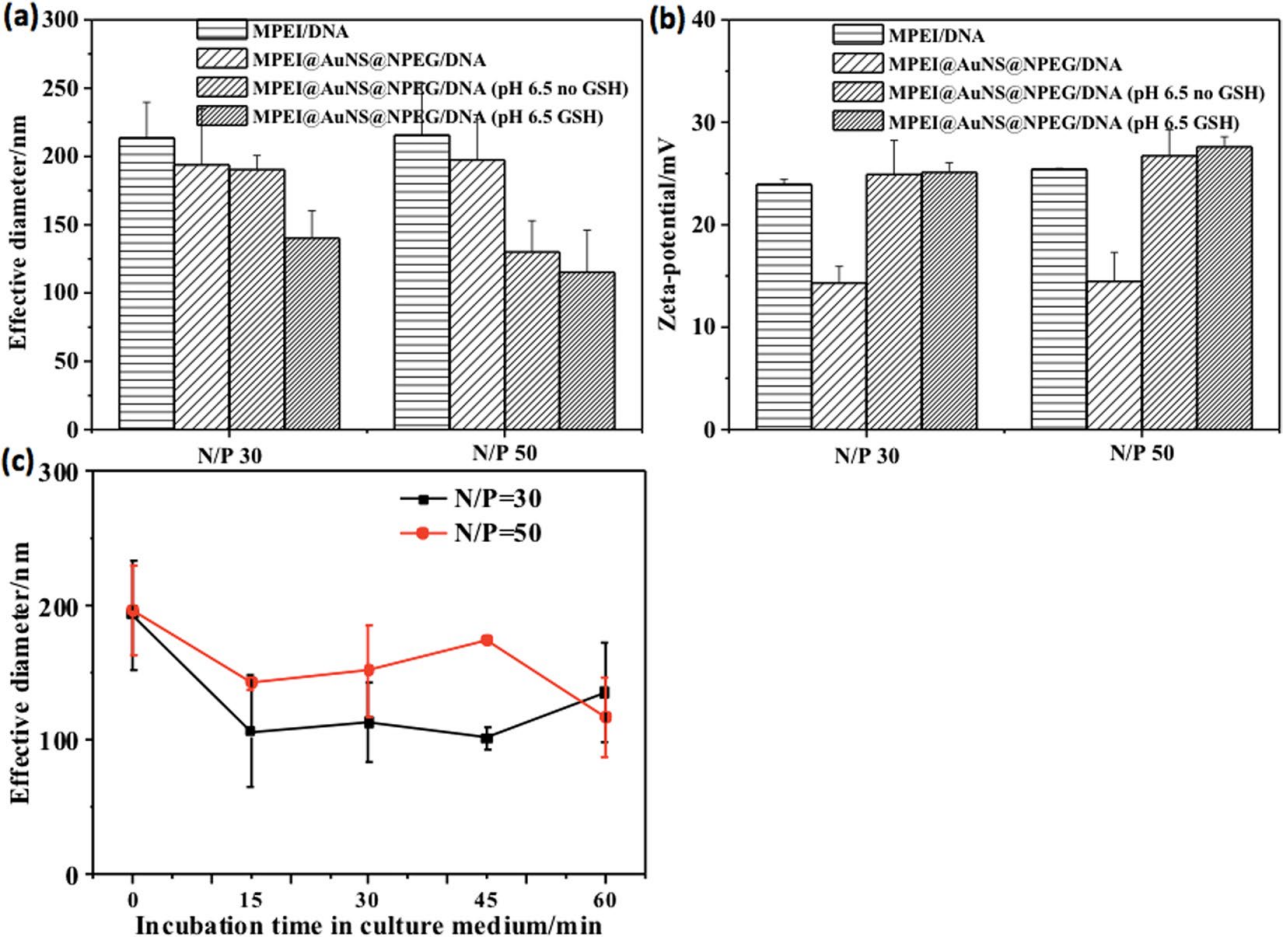
(**a**) Effective diameters and (**b**) zeta-potentials of the polyplexes at different N/P ratios; (**c**) Time-dependent diameters of MPEI@AuNS@NPEG/DNA polyplexes at N/P ratios of 30 and 50 in culture medium. (Error bar stood for S.D.).

The DNA condensation capability of the MPEI@AuNS@NPEG/DNA polyplexes was measured by the gel retardation assay^24^. At low N/P ratios, free DNA would electrophorese to positive electrode and be dyed by ethidium bromide (EtBr), which showed bright DNA band as shown in Fig. 4(a). However, while N/P ratio increased above 2, the free DNA band disappeared. This indicated all DNA were wrapped tightly by MPEI@AuNS@NPEG, with no free DNA left above N/P ratio of 2 (Fig. 4(a)). And the TEM images showed clearly that AuNSs were well-distributed in the spherical nanoparticles with diameters around 200 nm (Fig. 4(b,c)). This was consistent with the dynamic light scattering (DLS) data. As shown in Fig. 5(a), the diameters of MPEI@AuNS@NPEG/DNA at both N/P ratio of 30 and 50 were also about 200 nm. Also, with the shielding effect of NPEG, the zeta-potentials of MPEI@AuNS@NPEG/DNA polyplexes decreased a lot compared with MPEI/DNA (Fig. 5(b)). The NPEG shell could further stabilize the polyplexes in culture medium which was shown in Fig. 5(c). This further confirmed the successful assembly of MPEI@AuNS@NPEG/DNA.

Long term circulation relies on the existence of NPEG shell^25^. However, when gene vectors reached target tissue and cells, NPEG shell was expected to detach for high cellular uptake^26, 27^. Thus, in our study, pH- and GSH-responsive bonds were introduced into NPEG and MPEI respectively. To verify the stimuli-responsive property, the polyplexes were treated at pH of 6.5 and at the presence of GSH (10 mM). As shown in Fig. 5(b), after being incubated at pH of 6.5 for 30 min, zeta-potentials at both N/P ratios of 30 and 50 enhanced to the same level as MPEI/DNA. And the values did not increase after the further incubation in GSH. This indicated the whole detachment of NPEG shell at pH of 6.5. Also, AuNSs separated with the low-contrast polymers in TEM images and DLS showed the diameters decreased once being treated at pH of 6.5. All data mentioned above confirmed the stimuli-responsive effect of the polyplexes under the experimental conditions.

### Cytotoxicity assay

**Figure 6:**
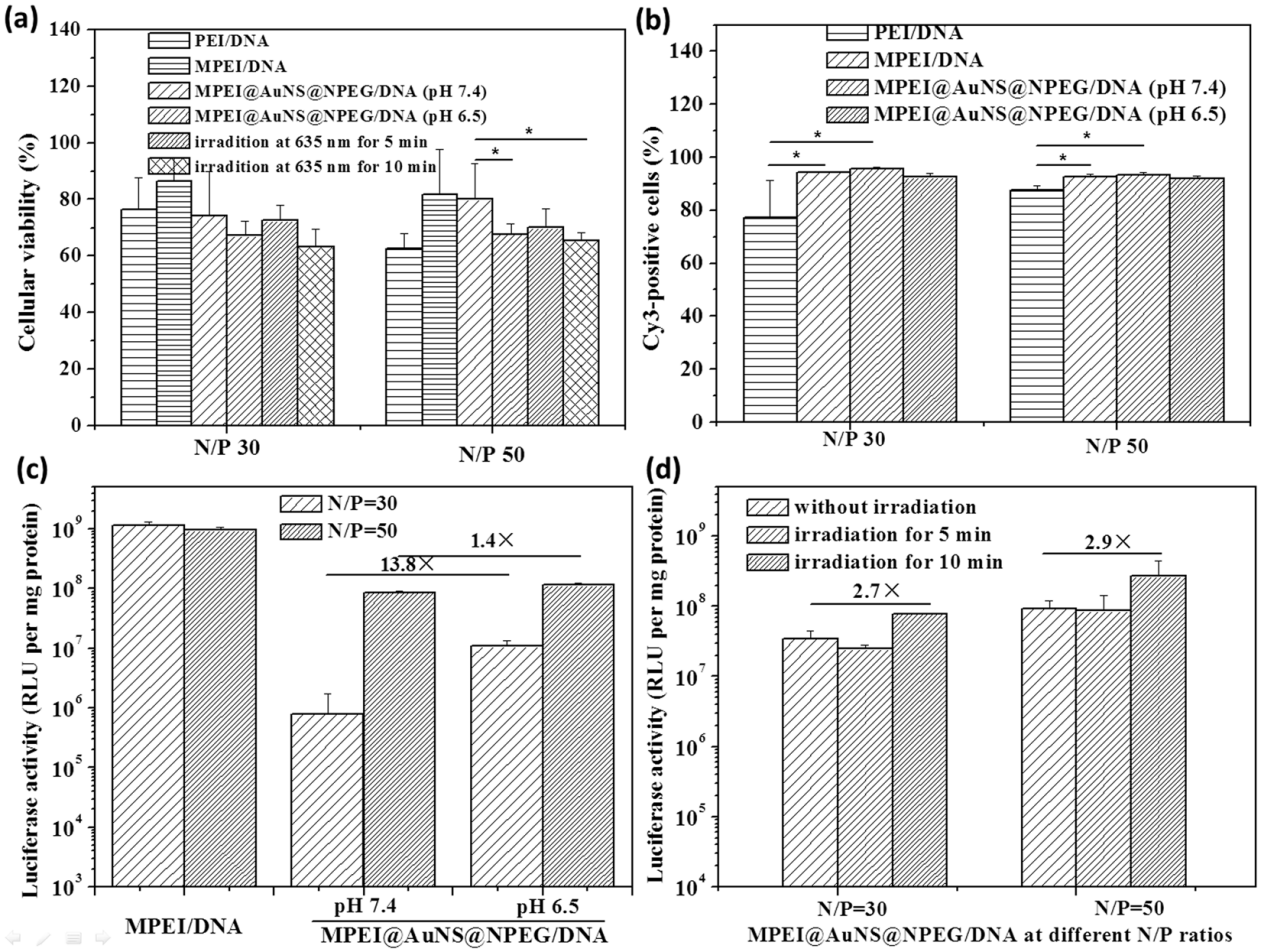
(**a**) Cellular cytotoxicity and (**b**) cellular uptake assays of different DNA polyplexes; (**c**) *In vitro* gene transfection of polyplexes as pH lowed; (**d**) *In vitro* gene transfection of polyplexes under light irradiation. (Error bar stood for S.D.; *Referred that p < 0.05).

Good cell compatibility is the prerequisite as gene vector^28^. The cytotoxicity of MPEI@AuNS@NPEG/DNA polyplexes was studied using MTT assay. In our experiment, cells were incubated at pH of 6.5 with MPEI@AuNS@NPEG/DNA polyplexes for 4 h before the replacement of fresh medium. To further study the light responsive properties, the cells were then exposed to light for 5 min and 10 min. Thus, cytotoxicity at pH of 6.5 and under light irradiation was assessed. What was shown in Fig. 6(a) indicated relatively low cytotoxicity for both MPEI/DNA and MPEI@AuNS@NPEG/DNA polyplexes at N/P ratios of 30 and 50. However, after being incubated at pH of 6.5 for 4 h, cellular viability decreased a little. This might ascribe to the low pH (pH 6.5) which detached PEG shell and exposed the cationic chains to agitate cell membrane more fiercely. As light irradiated for 10 min, there was no significant difference in cytotoxicity. Furthermore, the cytotoxicity of MPEI@AuNS@NPEG/DNA polyplexes at N/P of 30 and 50 was similar with no significant difference. In a word, the MPEI@AuNS@NPEG/DNA polyplexes showed relatively good cyto-compatibility at such high N/P ratios compared with PEI/DNA group at pH of 7.4.

### The cellular uptake efficiency of MPEI@AuNS@NPEG/DNA polyplexes

Based on data mentioned above, the NPEG shell detached due to the stimulus. Thus, the exposed positive charges would facilitate the electrostatic interaction with cell membrane. This might result in the enhancement of cellular uptake efficiency. To prove this, cellular uptake assays were implemented using HepG2 cells. It was unexpected to find that almost all MPEI@AuNS@NPEG/DNA polyplexes were uptook by cells, with cell uptake efficiency (ca. 90%) higher than that of PEI/DNA (ca. 80%) at both pH of 7.4 and 6.5 (Fig. 6(b)). This might be because the AuNSs in nanoparticles made it easy for polyplexes to contact with and adsorb on cell membrane. And this left no room for further enhancement of cellular uptake at pH of 6.5. Thus, the cellular uptake efficiency of MPEI@AuNS@NPEG/DNA polyplexes at pH of 6.5 had no significant difference with that at pH of 7.4, which also reached 90%.

### *In vitro* transfection efficiency

As was reported, the positive polyplexes could agitate cytomembrane which not only promoted cellular uptake but also facilitated endosome escape^29^. These two processes were the key points for gene vectors to enter cytoplasm for expression. In this way, we speculated that the exposure of positive charges after the detachment of NPEG at pH of 6.5 could promote gene expression. In this experiment, luciferase plasmid DNA pGL-3 was used for the quantitative analysis of gene transfection. The expression of luciferase defined as luciferase activity was detected by a luminometer and measured in relative light units (RLU). It was exciting to find that the low-pH (pH 6.5) treated groups had higher gene transfection efficiency, with 13.8 and 1.4 times higher at N/P ratios of 30 and 50 respectively than that at pH of 7.4 (Fig. 6(c)). Without the protection of NPEG, AuNSs tended to interact with other AuNSs when they assembled with CB[7]. This would lead to the aggregation of AuNSs and the red shifting of UV-Vis absorption (Fig. 3). Thus, attempts were made to research in the photo-thermal effects of AuNSs on gene transfection efficiency. As shown in Fig. 6(d), gene transfection efficiency of MPEI@AuNS@NPEG/DNA had no significant difference after 5 min light irradiation at 635 nm. However, as time extended to 10 min, about 2.7 and 2.9 times enhancement appeared for MPEI@AuNS@NPEG/DNA at N/P ratios of 30 and 50, respectively. These might be because the photo-thermal effects of AuNSs transformed light into heat after 10 min irradiation. These would accelerate the fluidity of cell membrane and were helpful for endosome escape or direct entry of gene into cytoplasm.

### Intracellular trafficking of MPEI@AuNS@NPEG/DNA polyplexes

**Figure 7:**
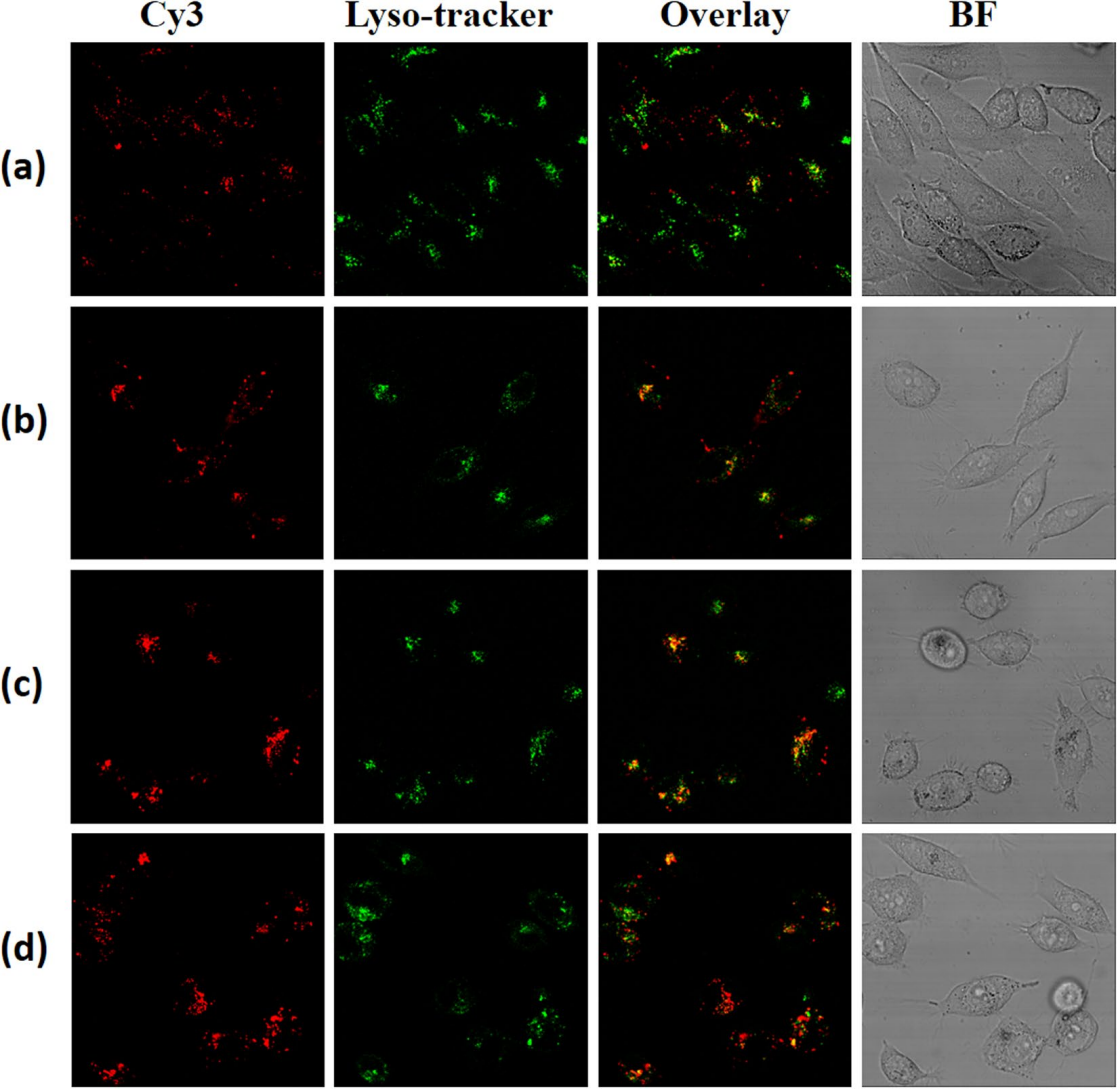
CLSM images of cells exposed to (**a**) PEI/Cy3-DNA, (**b**) MPEI/Cy3-DNA and MPEI@AuNS@NPEG/Cy3-DNA (**c**) without or (**d**) with light irradiation. The endosome were dyed green by LysoTracker® Green DND.

Encouraged by these results, intracellular fluorescent tracing was proceeded to find the exact mechanism for higher gene transfection efficiency by confocal laser scanning microscopy (CLSM)^11, 30^. Cy3-DNA and lyso-tracker green was used for better detection. Same as what was expected, red fluorescence of PEI/DNA separated clearly with the green endosome, indicating the excellent endosome escape ability of PEI. Also, MPEI/DNA without the shielding of NPEG could escape from endosome efficiently as shown in Fig. 7(b). However, once assembled with Np-CH=N-PEG, obvious yellow fluorescence was observed which illustrated that most of the MPEI@AuNS@NPEG/DNA polyplexes were still located in endosome. Nevertheless, when light at 635 nm was irradiated for 10 min, bright red dots were observed (Fig. 7(d)). This rightly explained the enhanced gene transfection after light exposure. The potential reason was that the photo-thermal effect of AuNSs could accelerate the fluidity or even disrupt cell membrane, which finally released gene vectors into cytoplasm. In a way, the introduction of photo-thermal effect revealed an effective way in the design of gene vector.

## Conclusions

In conclusion, host-guest interaction of CB[7] was utilized to easily link ligands onto CB[7]. By assembling with AuNSs, different segments such as PEG and PEI could be bound together. Then, via electrostatic interaction, it could condense DNA into 200 nm polyplexes with AuNSs well-distributing in the polyplexes. Also the pH- and GSH- detachment of NPEG shell solved the contradiction between stability and cellular uptake effectively. The gene polyplexes were found to be easily uptaken by cells. Most interestingly, the heated AuNSs could effectively agitate membrane once been irradiated at 635 nm for 10 min because of the photo-thermal effect of AuNSs. CLSM results indicated light irradiation might promote the direct entry of polyplexes into cytoplasm or the quick escape from endosome for high transfection efficiency. This provided a new way to exploit host-guest interaction in the design of drug/gene vector. By utilizing therapeutic genes instead of reported genes, synergistic effects of both gene therapy and photo-thermal effect might be obtained.

## Experimental Section

### Materials and Methods

Branched polyethylenimine (PEI, 25 kDa) was obtained from Sigma-Aldrich. Methoxypolyoxyethylene amine (mPEG-NH_2_, 5 kDa) and 2-Naphthylacetic acid were obtained from Aladdin (Shanghai, China). 4,4′-bipyridine, methyl iodide and potassium thioacetate were purchased from Sinopharm Chemical Reagent Co., Ltd. 1,10-dibromodecane was purchased from darui finechemical Co., Ltd. Potassium hexafluorophosphate (KPF_6_), n-Bu_4_NCl and 3,3′-Dithiodipropionic acid (DTPA) were obtained from Aladdin (Shanghai, China). All other reagents and solvents were of analytical grade and used as received without further purification.

Deoxyribonucleic acid (DNA, fish sperm, sodium salt) were obtained from AMRESCO for physiological measurements such as dynamic light scattering (DLS), zeta potential and transmission electron microscope (TEM). Cy3-labeled DNA (Cy3-DNA) was purchased from Sangon Biotech (Shanghai, China) for cell uptake determination and intracellular trafficking of polyplexes. Plasmid DNA pGL3 was obtained from Promega (USA) for gel retardation assay and gene transfection. 3-(4,5-dimethylthiAzl-2-yl)-2,5-diphenyltetrAzlium bromide (MTT) was obtained from Bio Basic Inc. Loading buffer was purchased from TakaRa Biotechnology (Dalian, China) Co.Ltd. 0.5 × TBE buffer was diluted from 4 × TBE buffer (0.36 M tris-boric acid, 8 mM elhylene diamine tetraacetic acid).

### Synthesis of N-Methyl-4,4′-bipyridinium Iodide (MV + )

4,4′-bipyridine (1.0 g, 6.4 mmol) was dissolved in 15 mL of dichloromethane (DCM). Then, methyl iodide (0.5 mL, 8.1 mmol) in DCM (5 mL) was added dropwise to the stirred solution. The mixed solution was stirred at room temperature overnight. The yellow product was filtered off and purified by recrystallization from methanol. ^**1**^ **H NMR (** ***d***_**3**_ **-MeCN):** ***δ*** **= 9.10 (d, 2 H), 8.79 (d, 2 H), 8.62 (d, 2 H), 8.05 (d, 2 H), 4.45 ppm (s, 3 H)**.

### Synthesis of N-(10-Mercaptodecyl)-N’-methyl-4,4’-bipyridinium Chloride (Mv-SH)

The obtained MV + (2 g, 6.7 mmol) and 1,10-dibromodecane (1.76 g, 8.7 mmol) were refluxed in methyl cyanide for 3 days. The precipitant was filtered and washed with warm methyl cyanide and dried under vacuum. Then the product (4.4 g, 7.4 mmol) and potassium thioacetate (1.3 g, 11.1 mmol) were taken into ethyl alcohol (100 mL) and refluxed for several hours. Then concentrated H_2_SO_4_ was added to obtain a pH = 1 solution and the solution was reflux for further 24 h. The black precipitant was filtered off. KPF_6_ (2.6 g, 16 mmol) was added to the filtrate. The resulting white/yellow precipitate was filtered off and washed with water and dried under vacuum. Then the dried product (0.58 g, 0.91 mmol) was dissolved in 15 mL of acetone. To this solution, n-Bu_4_NCl (1.06 g, 3.8 mmol) in 10 mL of acetone was added; the yellow/white precipitate (299 mg) was collected by centrifuge, washed by acetone, and dried under vacuum. The obtained Mv-SH was characterized by ^1^H NMR. ^**1**^ **H NMR (** ***d***_**4**_ **-MeOD):** ***δ*** = **8.42–9.28 (aromatic, 8H), 4.74 (t, 2H), 4.42 (s, 3H), 2.66 (t, 2H), 2.58 (t, 2H), 1.10–1.85 ppm (t, 14H)**.

### Synthesis of Mv-S-S-PEI (MPEI)

Firstly, DTPA grafted PEI (PEI-DTPA) was synthesized via condensation reaction between -COOH and -NH_2_ and confirmed by ^1^H NMR. In this way, -S-S- was introduced into PEI. Then, 140 mg of PEI-DTPA was dissolved in 2 mL phosphate buffer. At the presence of tris(2-carboxyethyl) phosphine (TCEP) (11 mg, 0.044 mmol), -S-S- was break and -SH was exposed. Meanwhile, 45 mg Mv-SH (0.11 mmol) was added and stirred for 2 h under the oxygen atmosphere. Finally, 1% H_2_O_2_ was added to make sure the complete reaction of -SH. The production was dialyzed for 4 days before lyophilization process. The final product was characterized by ^1^H NMR. ^**1**^ **H NMR (D**_**2**_ **O):** ***δ*** = **8.42–9.00 (aromatic, 8 H), 2.05–3.10 (m, -CH**_**2**_ **CH**_**2**_ **-NH-), 1.28–1.65 ppm (t, 14 H)**.

### Synthesis of Np-CH=N-PEG (NPEG)

4-hydroxybenzaldehyde was introduced into mPEG-NH_2_ via condensation reaction between -NH_2_ and CHO to prepare PEG-OH. Briefly, mPEG-NH_2_ (5 kDa, 0.43 g) was dissolved in 20 mL methanol and mixed with 4-hydroxybenzaldehyde (0.105 g). The solution was refluxed at 70 °C for 7 h and precipitated in ice ether. The resulted precipitate was washed and collected for vacuum drying. ^**1**^ **H NMR (CDCl**_**3**_ **):** ***δ*** = **7.00–7.80 (aromatic, 4H), 3.30–3.90 ppm (m, -CH**_**2**_ **CH**_**2**_ **-O-)**.

After the characterization of ^1^H NMR, the product was dissolved in 20 mL of anhydrous DCM. Then, 2-naphthylacetic acid, dicyclohexylcarbodiimide (DCC) and 4-dimethylaminopyridine (DMAP) were added with the mole ratio of 1(-OH):3(-COOH):2(DCC):0.1(DMAP). The solution was stirred at room temerature for 48 h for complete reaction. Then, filtration process was carried out. The filtrated solution was condensed and dialyzed for 3 days before lyophilization. The final product was confirmed by ^1^H NMR. ^**1**^ **H NMR (CDCl**_**3**_ **):** ***δ*** = **7.35–7.88 (aromatic, 11H), 3.30–3.90 ppm (m, -CH**_**2**_ **CH**_**2**_ **-O-**).

### MPEI@AuNS@NPEG/DNA polyplexes formation protocol

AuNSs were synthesized according to the previously literature^31^. Briefly, 1 ml of 10 mM HAuCl_4_ was diluted in 90 ml Milli-Q water and the solution was heated to boiling. To this boiling solution, 0.7 ml of 25 mM sodium citrate was added immediately. Then the solution was stirred till the color of the solution turned to wine red. These obtained AuNSs were observed by TEM and detected by UV-Vis spectrophotometer.

CB[7], MPEI and NPEG were firstly dissolved in Milli-Q water at certain concentration. Then, CB[7] solution was vortexed with MPEI and NPEG solution separately with the mole ratio of both Mv:CB[7] and Np:CB[7] at 1:1. To make sure the complete inclusion with Mv and Np, the solution was vortexed overnight after sonication for 30 min. MPEI@CB[7] solution and NPEG@CB[7] solution was mixed at mole ratio of 1:10 and at the same time, AuNSs solution was added. The mixed solution was vortexed vigorously for 1 min and incubated for 30 min. Thus, MPEI@CB[7] and NPEG@CB[7] would assemble on AuNSs surface through the interaction between CB[7] and AuNSs. The resulted solution was named MPEI@AuNS@NPEG solution.

After incubation for 30 min, the MPEI@AuNS@NPEG solution was then mixed with equal volume of DNA solution at certain N/P ratios. The resulted solution was vortexed for 1 min and incubated for 30 min before further assay. The amount of DNA was fixed for each experiment and N/P ratio was adjusted by changing the concentration of MPEI@AuNS@NPEG solution. The formed polyplexes were named MPEI@AuNS@NPEG/DNA. For all the polyplexes, N/P ratio referred to the mole ratio between protonatable nitrogen atoms of PEI and the phosphate groups of the DNA. For other polyplexes, similar process was carried out.

### Chemo-physical characterization of different polyplexes

CB[7] was reported to interact with AuNSs both sides which would cause the aggregation of AuNSs^4, 21^. However, when CB[7] formed inclusion with MPEI or NPEG, this would leave just one side of CB[7] interacting with AuNSs. Thus, MPEI or NPEG segments might be linked onto AuNSs. To prove it, digital photos and TEM images of AuNSs at the presence of CB[7], MPEI@CB[7] and MPEI@AuNS@NPEG were taken and their corresponding UV-Vis spectra were measured. Briefly, MPEI@CB[7] and NPEG@CB[7] solution were prepared as mentioned above. After that, the CB[7], MPEI@CB[7] and NPEG@CB[7] solution with the same amount of CB[7] was added into AuNSs solution respectively. The mixed solution was placed for 2 h before UV-Vis measurement. The DNA condensation capability of MPEI@AuNS@NPEG/DNA polyplexes was examined by gel retardation assay^1^. The polyplexes containing 450 ng pDNA were prepared based on the previous protocol, mixed with loading buffer (5:1 by volume) and electrophoresed at 100 V for 50 min. Then the gel was immersed in ethidium bromide solution (0.5 μg mL^−1^) for 30 min and observed by UV illuminator (Gel Doc, Bio-Rad, USA).

The sizes and zeta-potentials of MPEI@AuNS@NPEG/DNA polyplexes were measured by DLS. Measurement was performed on a Zetasizer Nano ZS90 (Malvern Inst. Ltd, UK) equipped with either a four-side clear cuvette for particle size analysis or a DTS 1060 C cell for zeta-potential measurement. For particle size analysis, the samples were carried out in 3 serial measurements at 25 °C (scattering angle 173°) and the zeta-potential measurements were performed in four times.

The morphology of MPEI@AuNS@NPEG/DNA polyplexes was observed by TEM (JEM-1200EX, NEC, Tokyo, Japan) operated at 80 kV. Briefly, 20 μL solution of the polyplexes at N/P ratios of 30 and 50 was dropped onto 200-mesh carbon-coated copper grid for 15 min. The process was repeated twice to obtain enough particles on the copper grid.

To verify if the -CH=N- bond of NPEG and -S-S- bond of MPEI could respond to pH and GSH stimuli respectively, the size, zeta-potential and morphology of MPEI@AuNS@NPEG/DNA polyplexes were measured after pre-treatment at pH of 6.5 and the presence of GSH. Briefly, the prepared MPEI@AuNS@NPEG/DNA solution was firstly adjusted to pH of 6.5 and incubated for 30 min before chemo-physical measurement. For GSH stimuli, the solution was firstly incubated at pH of 6.5 for 30 min. Then, GSH solution was added to obtain final GSH concentration of 10 mM.

The stability of MPEI@AuNS@NPEG/DNA polyplexes at pH of 7.4 was measured as follows. Briefly, MPEI@AuNS@NPEG/DNA polyplexes at N/P ratios of 30 and 50 were prepared. Then the solution was diluted by culture medium and the time-dependent particle sizes were monitored by Zetasizer Nano ZS90 (Malvern Inst. Ltd, UK).

### Cell culture

Human hepatoblastoma cell line (HepG2) was cultured in Dulbecco Modified Eagle Medium (DMEM) with 10% fetal bovine serum (FBS) and 1% penicillin-streptomycin. Cells were maintained under humidified air containing 5% CO_2_ at 37 °C.

### Cytotoxicity assay

Cell toxicity of different polyplexes was evaluated by MTT assay. Briefly, HepG2 cells were seeded in 96-well plates and cultured overnight to 80% cell confluence. Then the medium was replaced with fresh medium at pH of 7.4 or 6.5 and MPEI@AuNS@NPEG/DNA polyplexes were added. For light irradiation groups, 635 nm light was exposed to cells for 5 min or 10 min after 4 h incubation. After incubation for 48 h, the medium was replace by fresh medium and 20 μL of MTT (5 mg mL^−1^) was added. The cells were incubated for 4 h at 37 °C. Then the medium was replaced with 200 μL of dimethyl sulphoxide (DMSO) to resolve formazan. The absorbance at 570 nm was measured by microplate reader (550, Bio-Rad, USA). All experiments were performed in quintuplicate. “Golden standard” transfection agent PEI_25k_/DNA and MPEI/DNA were used as control.

### The cellular uptake efficiency of MPEI@AuNS@NPEG/DNA polyplexes

HepG2 cells were seeded into 24-well plates at a density of 5 × 10^4^ cells per well and cultured for 24 h. Then, culture medium was changed with fresh culture medium at pH of 6.5 and 7.4 respectively. MPEI@AuNS@NPEG/Cy3-DNA polyplexes were added and incubated for 4 h. The cells were washed three times with PBS, trypsinized and analyzed by flow cytometry. PEI_25k_/DNA and MPEI/DNA were used as control. All experiments were performed in triplicate.

### *In vitro* transfection efficiency

HepG2 cells were seeded in 24-well plates at a density of 5 × 10^4^ cells per well. pGL3-DNA was used for transfection assay. After incubating overnight, the medium was replaced with fresh complete medium at pH of 6.5 and 7.4. MPEI@AuNS@NPEG/DNA polyplexes at N/P ratios of 30 and 50 were added and incubated for 4 h before the replacement of fresh medium. After incubating for another 44 h, luciferase activity was measured in relative light units (RLU) using luminometer. Results were normalized to total cell protein as determined using a KEYGEN BCA protein assay. All experiments were performed in triplicate.

To see if light irradiation could affect gene transfection efficiency, 635 nm light was exposed to cells. Briefly, after 4 h incubation with MPEI@AuNS@NPEG/DNA polyplexes at pH of 6.5, the medium was replaced with fresh culture medium and 635 nm light was irradiated for 5 min and 10 min, respectively. Total 48 h later, luciferase activity was measured as described previously. All experiments were performed in triplicate.

### Intracellular trafficking of MPEI@AuNS@NPEG/DNA polyplexes

5 × 10^4^ HepG2 cells were seeded into glass base dishes and cultured for 24 h. Fresh complete medium containing polyplexes assembled with Cy3-DNA at N/P ratio of 50 was added. After 4.5 h incubation, the medium was replaced with fresh medium and maintained for 12 h. For light irradiation group, 635 nm light was immediately exposed to cells for 10 min. Then, the lysosome was stained in serum free medium with LysoTracker® Green DND according to the manufacture’s protocol. After 0.5 h of dyeing, the dishes were washed with PBS four times. Intracellular distribution of the polyplexes was then observed by confocal laser scanning microscope (CLSM, Leica TS SP5, Germany). PEI_25k_/DNA and MPEI/DNA were used as control.
